# Supplementary material for: Thermodynamic determination of the equilibrium first-order phase-transition line hidden by hysteresis in a phase diagram
Source: Sci Rep. 2023 Apr 27;13:6876. doi: 10.1038/s41598-023-33816-6 (PMC10140377; doi:10.1038/s41598-023-33816-6)
Supplement: Supplementary file 1 — Supplementary Information. [file 41598_2023_33816_MOESM1_ESM.pdf]

Supplementary Information for
Thermodynamic determination of the equilibrium first-order phase-transition line hidden by hysteresis in a phase diagram

Keisuke Matsuura,[1,*] Yo Nishizawa,[2] Markus Kriener,[1] Takashi Kurumaji,[3]
Hiroshi Oike,[1,2,4] Yoshinori Tokura,[1,2,5] and Fumitaka Kagawa[1,2,6,†]

[1] *RIKEN Center for Emergent Matter Science, Wako 351-0198, Japan*

[2] *Department of Applied Physics and Quantum-Phase Electronics Center (QPEC), University of Tokyo, Tokyo 113-8656, Japan*

[3] *Department of Advanced Materials Science, University of Tokyo, Kashiwa 277-8561, Japan*

[4] *PRESTO, Japan Science and Technology Agency (JST), Kawaguchi 332-0012, Japan*

[5] *Tokyo College, University of Tokyo, Tokyo 113-8656, Japan*

[6] *Department of Physics, Tokyo Institute of Technology, Tokyo 152-8551, Japan*

[*] E-mail:keisuke.matsuura@riken.jp

[†] E-mail:kagawa@phys.titech.ac.jp

Supplementary Note 1: Application of the Maxwell relation to a broadened first-order phase transition

In this section, we consider the validity of the application of the Maxwell relation, $\left(\frac{\partial S}{\partial H}\right)_T = \left(\frac{\partial M}{\partial T}\right)_H$, across an FOT. Below, we compare the two cases shown in Supplementary Fig. 1: (i) an ideal FOT accompanied by the discontinuity in S and M and (ii) a broadened FOT accompanied by no such discontinuity. As mentioned in the main text, for the case of an ideal field-induced FOT, the mathematic basis of the validity of the Maxell relation is lost at the ideal transition field H_c, where S and M are not differentiable. However, a field-induced FOT is sometimes experimentally observed as a broadened transition due to a two-phase mixture. The magnetic-field region in which the broadened FOT occurs is denoted by H_1 and H_2 ($H_1 < H_c < H_2$). In this case, the discontinuous change at H_c for the case of the ideal FOT is replaced with a continuous change in S and M of the whole system ranging from H_1 to H_2, as shown in Supplementary Figs. 1**b** and 1**c**. S and M are differentiable throughout the H range; hence, the mathematic basis of the Maxell relation is recovered. A question of interest is whether the integration of $\left(\frac{\partial S}{\partial H}\right)_T = \left(\frac{\partial M}{\partial T}\right)_H$ with respect to H across the broadened FOT provides the correct value of ΔS^*, which is the entropy discontinuity for the case of the ideal FOT. In the following analysis, for simplicity, any hysteresis effect is neglected.

To analytically consider this issue, we introduce $S_{\alpha/\beta}$ and $M_{\alpha/\beta}$, which describe the entropy and magnetization of the α (or β) phase, respectively, as functions of T and H. We introduce $S^{'}$ and $M^{'}$, which describe the entropy and magnetization of the whole system for the case of the broadened FOT, respectively. $S_{\alpha/\beta}$ and $S^{'}$ differ only in the transition region. Hence, we set the boundary conditions as follows:

$$\begin{aligned} S^{'}(H_1,T) &= S_\alpha(H_1,T), \\ S^{'}(H_2,T) &= S_\beta(H_2,T), \\ M^{'}(H_1,T) &= M_\alpha(H_1,T), \\ M^{'}(H_2,T) &= M_\beta(H_2,T) \end{aligned} \tag{S1}$$

In the case of the ideal FOT, the entropy of phase β at (H_2, T) is given as follows:

$$
\begin{aligned}
S_\beta(H_2,T) &= S_\alpha(H_1,T) + \int_{H_1}^{H_c} \left(\frac{\partial S_\alpha}{\partial H}\right)_T dH + \Delta S^* + \int_{H_c}^{H_2} \left(\frac{\partial S_\beta}{\partial H}\right)_T dH \\
&= S_{\mathrm{A}}(H_1,T) + \int_{H_1}^{H_c} \left(\frac{\partial M_\alpha}{\partial T}\right)_H dH + \Delta S^* + \int_{H_c}^{H_2} \left(\frac{\partial M_\beta}{\partial T}\right)_H dH, \qquad \text{(S2)}
\end{aligned}
$$

where the contribution of the entropy discontinuity, ΔS^*, is explicitly considered. For the broadened FOT, in contrast, $S^{'}$ and $M^{'}$ are differentiable throughout the H region; hence, $S^{'}(T,H_2)$ are obtained by simply integrating $\left(\frac{\partial S}{\partial H}\right)_T$ from H_1 to H_2

$$
\begin{aligned}
S^{'}(H_2,T) &= S^{'}(H_1,T) + \int_{H_1}^{H_2} \left(\frac{\partial S^{'}}{\partial H}\right)_T dH \\
&= S^{'}(H_1,T) + \int_{H_1}^{H_2} \left(\frac{\partial M^{'}}{\partial T}\right)_H dH \qquad \text{(S3)}
\end{aligned}
$$

Given the boundary conditions of $S^{'}$, we obtain the following:

$$
\begin{aligned}
\Delta S^{'} \equiv \int_{H_1}^{H_2} \left(\frac{\partial M^{'}}{\partial T}\right)_H dH &= \int_{H_1}^{H_c} \left(\frac{\partial M_\alpha}{\partial T}\right)_H dH + \Delta S^* + \int_{H_c}^{H_2} \left(\frac{\partial M_\beta}{\partial T}\right)_H dH \\
&\geq \Delta S^* \qquad \text{(S4)}
\end{aligned}
$$

This equation indicates that $\int_{H_1}^{H_2} \left(\frac{\partial M^{'}}{\partial T}\right)_H dH$ across the transition region tends to result in an entropy change, $\Delta S^{'}$, larger than the ideal value, ΔS^* (see Fig. S1**b**). When the transition width, $H_2 - H_1$, is sufficiently narrow, the contributions of $\int_{H_1}^{H_c} \left(\frac{\partial M_\alpha}{\partial T}\right)_H dH$ and $\int_{H_c}^{H_2} \left(\frac{\partial M_\beta}{\partial T}\right)_H dH$ become small; hence, $\int_{H_1}^{H_2} \left(\frac{\partial M^{'}}{\partial T}\right)_H dH \approx \Delta S^*$. Nevertheless, for a nearly ideal FOT, $\left(\frac{\partial M^{'}}{\partial T}\right)_H$ becomes delta-function-like, and care should be taken regarding errors that may occur during numerical integrations.

Lastly, we comment on the hysteresis effect. Due to the phase coexistence, $\left(\frac{\partial M}{\partial T}\right)_H$ data obtained from isothermal $M(H)$ curves often leads to the experimental artifacts in the calculation of the isothermal entropy change across an FOT[S1,S2,S3,S4]. In the two-phase coexistence region, the dependency on a history in a phase diagram complicates the correct $\left(\frac{\partial M}{\partial T}\right)_H$ data acquisition; thus, integrating the Maxwell relation would cause even unphysical isothermal entropy change, such as a sharp peak in the temperature dependence of the isothermal entropy change[S1,S2,S3,S4]. Therefore, we avoided the data analysis in the two-phase coexistence region (see Fig. 2**b**). As mentioned in the main text, the present analyis can avoid the problems that are often caused by the two-phase coexistence.

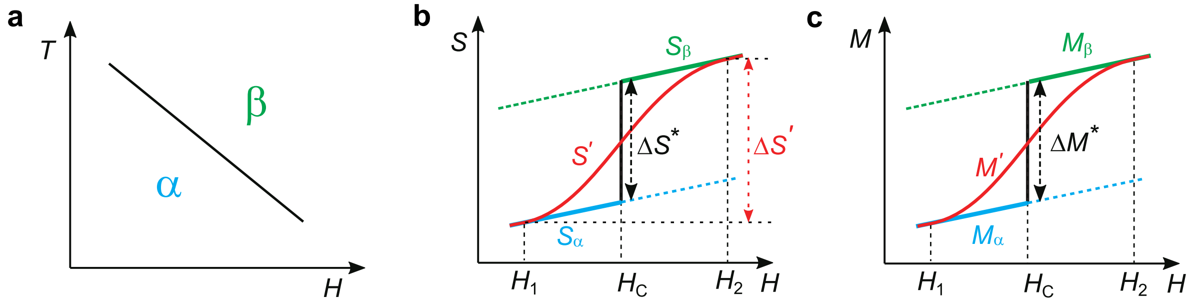

Supplementary Figure 1 | Comparison of ideal and broadened first-order phase transitions. **a** Schematic thermal-equilibrium phase diagram of the considered model. The α and β phases neighbor across a first-order transition line. **b,c** Isothermal entropy (**b**) and magnetization (**c**) curves.

Supplementary Note 2: Isothermal magnetization curves

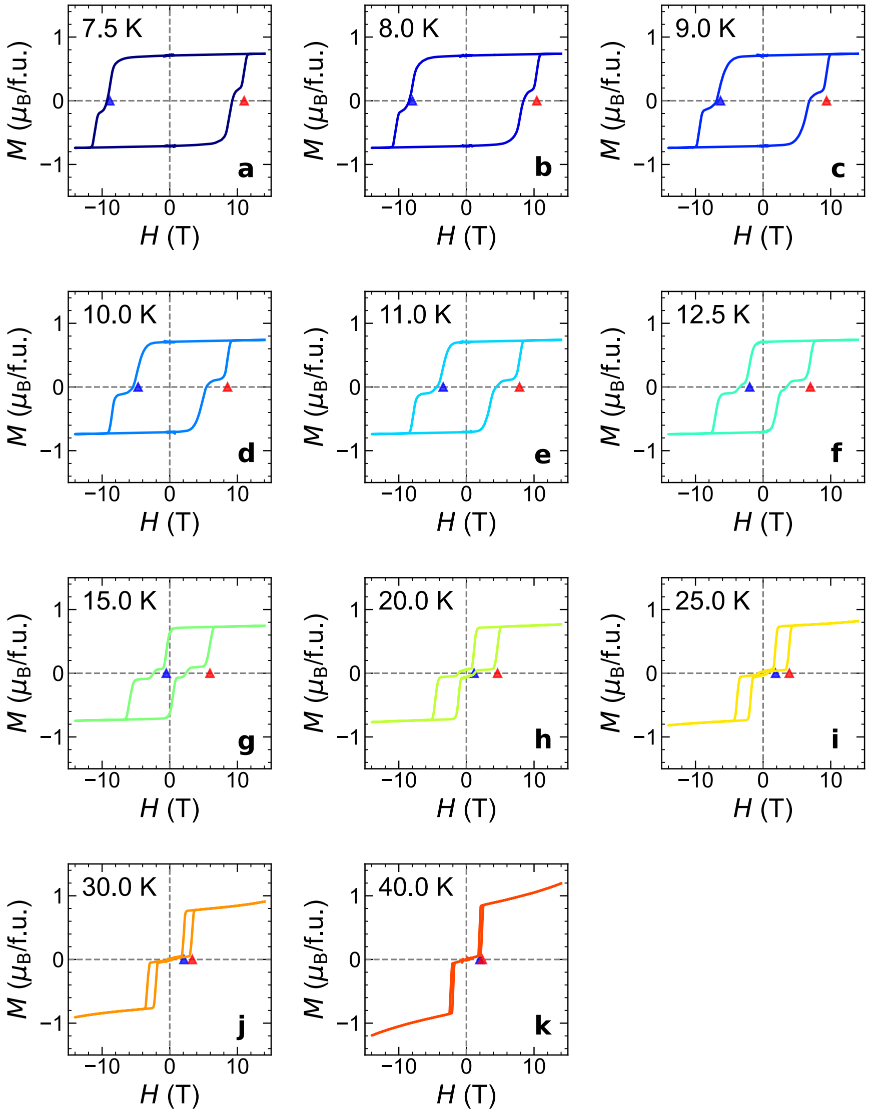

Supplementary Figure 2 | Isothermal magnetization curves of ($Fe_{0.95}Zn_{0.05}$)$_2$$Mo_3O_8$. a–k The isothermal magnetization curves of ($Fe_{0.95}Zn_{0.05}$)$_2$$Mo_3O_8$ between +14 T and -14 T at 7.5, 8.0, 9.0, 10.0, 11.0, 12.5, 15.0, 20.0, 25.0, 30.0, and 40.0 K, respectively. The transition from the AFM to the FRI phases and from the FRI to the AFM phases are indicated by red and blue triangles, respectively.

Supplementary Note 3: Field-warming data at 2 T after zero field cooling

Supplementary Fig. 3 compares the M–T curve at 2 T under field cooling and that under field warming after zero-field cooling (i.e., 2 T was applied after zero-field cooling to the lowest temperature, followed by warming at 2 T). These two curves agree with each other, indicating that the lowest-temperature magnetic state under field cooling is the AFM single phase.

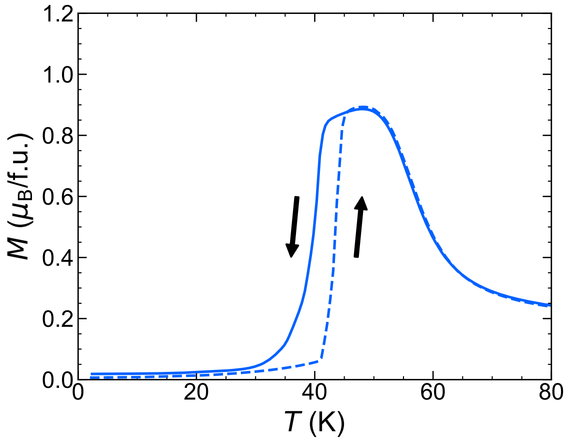

Supplementary Figure 3 | Comparison of the magnetization-temperature curves at 2 T under field cooling and under field warming after zero-field cooling. The solid and dashed lines indicate field cooling and zero-field cooling, respectively.

Supplementary Note 4: Extrapolation of the specific heat data below 2 K

A general analytic problem in converting the specific-heat data to the absolute entropy lies in the method of extrapolating the data to zero temperature, where the entropy is supposed to be zero according to the third law of thermodynamics. We find that the present $c_p(T)$ are nearly proportional to T^3 below 10 K (see solid lines in Supplementary Figs. 4**a-i**). A previous study has also reported that thermal transport is likely dominated by phonon-like excitations[S5]. The fitting coefficients of different magnetic fields are between 4.4×10^{-4} and 4.8×10^{-4} (J(mol·K^4)$^{-1}$). The specific heat in the Debye model in the sufficiently low-temperature region is given by

$$c_{\mathrm{D}} = \frac{12\pi^4}{5} n N_{\mathrm{A}} k_{\mathrm{B}} \left(\frac{T}{\Theta_D}\right)^3 \propto T^3, \tag{S7}$$

where n, N_{A}, k_{B}, Θ_{D} are the number of atoms per formula unit, Avogadro constant, Boltzmann constant, and Debye temperature, respectively[S6]. The Debye temperature Θ_{D} estimated from these coefficients of T^3 is approximately 380 K. In the present case, $\Theta_{\mathrm{D}}/50$ is ≈ 7.6 K, validating our T^3 analysis near 2 K. Thus, the $c_p(T)$ below 2 K at each magnetic field can be extrapolated to zero temperature by using T^3-fitting. After the extrapolation, we obtained $S_H(T)$ data of H = 0, 0.1, 1, 2, 3, 4, 5, 6, and 7 T from 0 K, as shown in Figs. 3**b–j** in the main text and Supplementary Fig. 4.

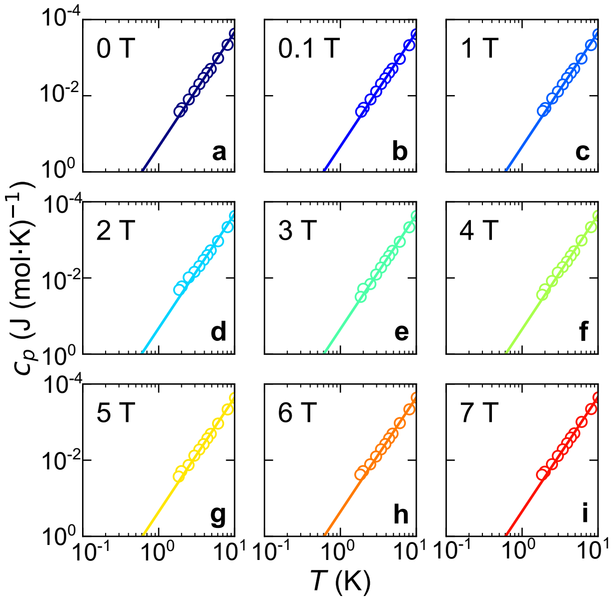

Supplementary Figure 4 | Extrapolation of the specific heat data below 2 K. a–i The data at 0, 0.1, 1, 2, 3, 4, 5, 6, and 7 T, respectively. Solid lines represent the fitting of $\propto T^3$. Fittings are performed using the data below 5 K.

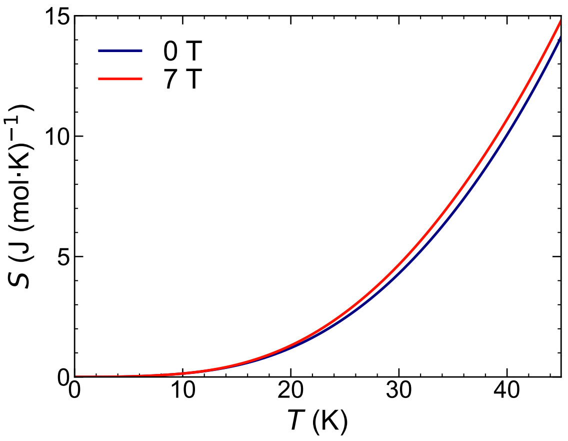

Supplementary Figure 5 | The temperature dependencies of the entropies in AFM (0 T; black) and FRI (7 T; red) phases.

Supplementary Note 5: $\frac{dM}{dT}$–T curves in the whole temperature range

In the derivation of isothermal $\left(\frac{\partial S}{\partial H}\right)_T$ curves via the Maxwell relation, the $\frac{dM}{dT}$–T data indicated by solid lines in Fig. 4**a** were used for the analysis. For the readers who are interested in the whole $\frac{dM}{dT}$–T data, Supplementary Fig. 6 shows the $\frac{dM}{dT}$–T data above 50 K. The measurements were done by field-cooling procedures.

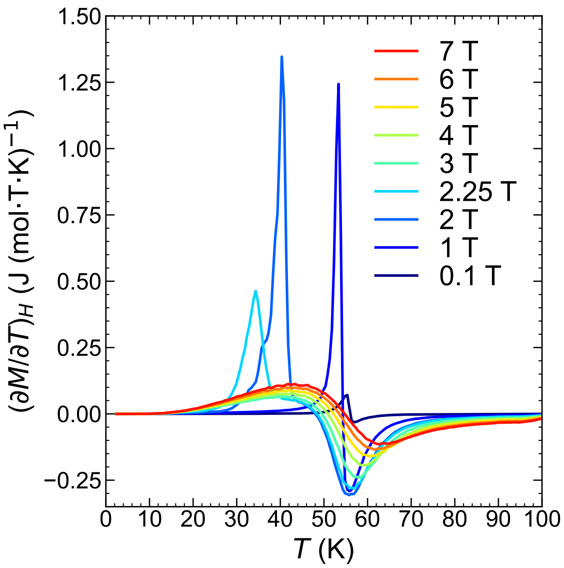

Supplementary Figure 6 | $\frac{dM}{dT}$–T curves measured upon decreasing temperature.

Supplementary Note 6: Verification of the equilibrium first-order transition line

To verify whether the obtained equilibrium FOT line is physically valid, we aim to reveal the equilibrium phase at 2.3 T and 10 K. At 10 K, the equilibrium phases at 0 and 6 T are obviously the AFM and FRI phases. However, this issue is more subtle for 2.3 T because the equilibrium phase may be the FRI phase if the midpoint line represents the equilibrium FOT line more accurately than the line we determined. In this section, focusing on the idea that metastable state relaxation occurs toward the most stable state, we discuss the equilibrium phase at 2.3 T and 10 K.

The relaxation measurements are performed at three points indicated by the black points in the inset in Supplementary Fig. 7. The initial states with $M(0)$ are prepared by cooling the sample from 100 to 10 K in an applied field of 2.3 T, and then the magnetic field is set to the respective target fields. As shown in Supplementary Fig. 7, the magnetization measured in 0 T decreases as a function of time, whereas it increases in 6 T; these findings are expected from the phase diagram. The magnetization at 2.3 T gradually decreases with time, demonstrating that the system relaxes toward the AFM phase, as in the case of 0 T. Although the change in the macroscopic magnetization is small and less than 0.05% within 10^4 s, this behaviour is difficult to explain if one considers the midpoint line as the equilibrium FOT line because this field position is assigned to the FRI phase in this case. Thus, we conclude that the line we determined is physically valid as the equilibrium FOT line, unlike the midpoint line.

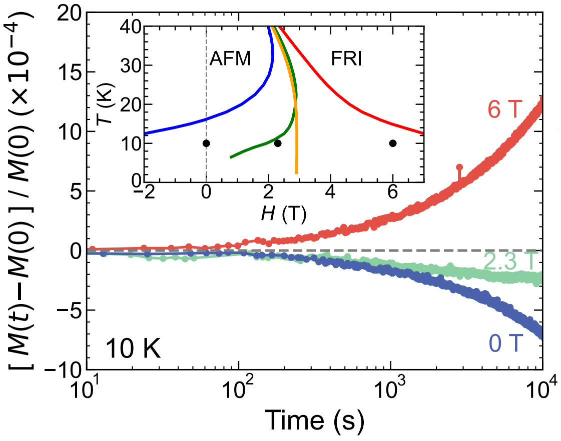

Supplementary Figure 7 | Verification of the equilibrium first-order transition line from the time dependence of mangetizaiton. Time dependence of the net magnetization $M(t) - M(0)$ normalized to the initial magnetization $M(0)$. The temperature was stabilized at 10 K after field cooling in 2.3 T from 100 K. Inset: Phase boundary obtained in this work. Red, blue, green, and orange lines represent the AFM to the FRI hysteresis lines, the FRI to the AFM hysteresis lines, the midpoint line between the red and blue lines, and the equilibrium FOT line, respectively. The relaxation measurements were performed at positions, as indicated by black points.

Supplementary References

S1. Liu, G., Sun, J., Shen, J., Gao, B., Zhang, H., Hu, F. & Shen, B. Determination of the entropy changes in the compounds with a first-order magnetic transition. *APL* **90**, 032507 (2007).

S2. Tocado, L., Palacios, E., & Burriel, R. Entropy determinations and magnetocaloric parameters in systems with first-order transitions. *J. Appl. Phys.* **105**, 093918 (2009).

S3. Amaral, J. & Amaral, V. On estimating the magnetocaloric effect from magnetization measurements. *J. Magn. Magn. Mater.* **322**, 1552–1557 (2010).

S4. Caron, L., Ou, Z. Q., Nguyen, T. T., Cam Thanh, D. T., Tegus, O., Brück, E. On the determination of the magnetic entropy change in materials with first-order transitions. *J. Magn. Magn. Mater.* **321**, 3559 (2009).

S5. Ideue, T., Kurumaji, T., Ishiwata, S. & Tokura, Y. Giant thermal Hall effect in multiferroics. *Nat. Mater.* **16**, 797-802 (2017).

S6. Ashcroft, N. W. & Mermin, N. D. *Solid State Physics* (Saunders College, 1976).
